# An investigation of the correlation between the S-glutathionylated GAPDH levels in blood and Alzheimer’s disease progression

**DOI:** 10.1371/journal.pone.0233289

**Published:** 2020-05-29

**Authors:** Chen Wei Tsai, Chia Fan Tsai, Kuan Hung Lin, Wei Jung Chen, Muh Shi Lin, Cho Chen Hsieh, Chai Ching Lin

**Affiliations:** 1 Department of Biotechnology and Animal Science, College of Bioresources, National Ilan University, Yilan City, Taiwan; 2 Department of Psychiatry, Taipei Veterans General Hospital, Taipei, Taiwan; 3 Department of Neurology, Taiwan Adventist Hospital, Taipei, Taiwan; 4 Department of Surgery, Kuang Tien General Hospital, Taichung, Taiwan; 5 Department of Biotechnology, College of Medical and Health Care, Hung Kuang University, Taichung, Taiwan; 6 Department of Health Business Administration, College of Medical and Health Care, Hung Kuang University, Taichung, Taiwan; 7 Dr. Power Stem International Group Co., Ltd, Taiwan; Massaschusetts General Hospital and Harvard Medical School, UNITED STATES

## Abstract

Alzheimer’s disease (AD) is a progressive neurodegenerative disease characterized by two aggregates, namely, amyloid-β (Aβ) plaques and neurofibrillary tangles (NFTs) of hyperphosphorylated tau protein (tau-p), which are released into the blood in a very small amount and cannot be easily detected. An increasing number of recent studies have suggested that S-glutathionylated glyceraldehyde 3-phosphate dehydrogenase (GAPDH) is highly correlated with Aβ in patients with AD and that S-glutathionylated GAPDH plays a role as a proapoptotic factor in AD. We found that S-glutathionylated GAPDH is abundant in the blood of AD patients, which is unusual because S-glutathionylated GAPDH cannot exist in the blood under normal conditions. The aim of this study was to further explore the correlation between the S-glutathionylated GAPDH levels in blood plasma and AD progression. As controls, we recruited 191 people without AD, which included 111 healthy individuals and 37 patients with depression and insomnia, in the psychosomatic clinic. Moreover, 47 patients with AD (aged 40–89 years) were recruited at the neurology clinic. The blood S-glutathionylated GAPDH levels in the AD patients were significantly (p < 0.001) higher (752.7 ± 301.7 ng/dL) than those in the controls (59.92 ± 122.4 ng/dL), irrespective of gender and age. For AD diagnosis, the criterion blood S-glutathionylated GAPDH level > 251.62 ng/dL exhibited 95.74% sensitivity and 92.67% specificity. In fact, the individuals aged 70–89 years, namely, 37 patients from the psychosomatic clinic and 42 healthy individuals, showed significant blood S-glutathionylated GAPDH levels (230.5 ± 79.3 and 8.05 ± 20.51 ng/dL, respectively). This finding might indicate neurodegenerative AD progression in psychosomatic patients and suggests that the degree of neuronal apoptosis during AD progression might be sensitively evaluated based on the level of S-glutathionylated GAPDH in blood.

## Introduction

The majority of dementia cases can be diagnosed as AD [1], which is classified as a neurodegenerative disorder whose cause and progression are poorly understood. The pathogenesis of AD might involve amyloid plaques and tangles in the brain and short-term memory loss, which is defined as difficulty remembering recent events [2]. An easily detected biomarker of AD risk to evaluate the level of neuronal apoptosis in the brain more than 10–15 years prior to early-stage AD can potentially be incorporated into routine health examinations for earlier prevention of brain deterioration. In general, AD is diagnosed when patients present symptoms of memory loss at clinics, and most of these AD diagnoses are based on the results of clinical interviews and neuropsychological scores, such as the Cognitive Abilities Screening Instrument (CASI) [3], the Short Portable Mental State Questionnaire (SPMSQ) [4], the National Institute of Neurological and Communicative Disorders and Stroke–Alzheimer’s Disease and Related Disorders Association (NINCDS-ADRDA) [5], and the Mini-Mental State Examination (MMSE) [6]. In fact, these scores do not accurately detect the early stages of dementia and do not distinguish between AD and vascular dementia. Furthermore, some atypical cases of AD are diagnosed based on imaging studies comprising computed tomography (CT), magnetic resonance imaging (MRI), or the analysis of biomarkers in cerebrospinal fluid [7] obtained via lumbar puncture (LP). Based on the recommendations provided by Swedish national guidelines, CT can be performed as a basic workup. Thus, MRI and LP are selected when an extended workup is needed [8]. Recently developed methods for AD diagnosis include the examination of Aβ and tau-p protein in blood plasma through immune-magnetic reduction (IMR) biotechnology, which exhibits 85% accuracy [9, 10]. However, with the exception of the neuropsychological scores, the described tests are costly and cannot be easily incorporated into routine health examinations. An easier and less expensive blood marker might be a good indicator of early stages of neurodegeneration in AD.

We investigated the correlation between the blood level of S-glutathionylated GAPDH and AD because some studies have demonstrated that the oxidative forms of GAPDH are aggregated with Aβ and tau-p plaques and are abundantly found in the pathological brains of patients with AD [11, 12]. The oxidative dysfunction of GAPDH, such as its dysfunction due to S-nitrosylation [11, 13] and S-glutathionylation [12], has been identified in the AD hippocampus, and S-glutathionylated GAPDH, which is found at 7-fold higher levels in the brains of AD patients compared with control individuals, exhibits the strongest relationship with AD [11]. The levels of SNO GAPDH exhibited 2-fold changes in the brains of AD patients compared with controls [12]. Thus, these dysfunctional forms of GAPDH might significantly contribute to the loss of neuronal function and neurodegeneration observed in AD brains [11, 12].

The oxidative dysfunction of GAPDH might be highly related to the oxidation of ubiquitin carboxyl terminal hydrolase-1 (UCH-L1) [14] to induce protein aggregation, neuronal apoptosis, neuronal dysfunction, and neurodegenerative diseases. Moreover, oxidative GAPDH protein, which accumulates in the brains of patients with AD, colocalizes and specifically interacts with the Aβ precursor protein (APP) [15, 16], Aβ (1–42) [17, 18], NFTs [19, 20], and paired helical filament-tau (PHF-tau) [21, 22].

All GAPDH isozymes in the brains of patients with AD are altered through oxidation during neuronal apoptosis [23]. It has been confirmed that GAPDH, when it accumulates in mitochondria to alter the permeabilization of the mitochondrial membrane, has the ability to induce cell apoptosis and can thus be considered a proapoptotic factor [24]. Furthermore, oxidative GAPDH plays an apparent role in AD-related apoptotic cell death [25]. Previous findings have strongly suggested that oxidative GAPDH is a pro-apoptotic protein and that its oxidative dysfunction induces the transcription of genes that mediate cell death through AD-related apoptotic processes [26, 27].

In addition to its best-known role in glycolysis [28], the multifunctional GAPDH protein also participates in the maintenance of iron metabolism and DNA integrity [29], membrane trafficking [30], histone biosynthesis [31, 32], receptor-mediated cell signalling [33, 34], and other processes. Initially, GAPDH exists mainly in the cytoplasm as a homologous tetramer (approximately 150 kDa) and in other locations, such as the membrane, polysome, endoplasmic reticulum, and Golgi [35, 36]. During cell proliferation, GADPH is located in the nucleus as a monomer (approximately 37 kDa) [37, 38].

However, Aβ neurotoxicity is able to promote GAPDH disulphide formation [39] and its nuclear translocation and pro-apoptotic action [40]. In this study, we investigated whether S-glutathionylated GAPDH is released from apoptotic neurons to reflect the neurodegenerative situation in AD brains.

## Materials and methods

### Ethics statement

This research project was performed in accordance with the latest version of the Declaration of Helsinki, and the human plasma sample data were collected from clinical trials. The study subjects provided written informed consent at the time of plasma sample and data collection and completed questionnaires approved by the Institutional Review Board (IRB) at each participating site. This study was reviewed and approved by the IRB of Taipei Veterans General Hospital (TVGH) (IRB No. 2013-04-044B, 2013/5/7-2014/5/6) and subsequently by the IRB of Taiwan Adventist Hospital (TAH) (IRB No. 108-E-10, 2019/3/29-2020/3/28).

### Collection of human plasma samples in clinical trials

The AD patients were selected through diagnostic determinations based on the results from clinical interviews, physical examinations, neuropsychological testing, laboratory findings and image investigations (CT and/or MRI) at a clinical consensus meeting. An AD diagnosis was made according to the criteria for probable AD detailed by the National Institute of Neurological and Communicative Disorders and Stroke-Alzheimer’s Disease and Related Disorders Association (NINCDS-ADRDA) [5].

If a patient with AD was unable to understand the consent conditions, a responsible family member provided consent. We recruited 191 people aged 20–89 years who served as the controls. Thirty-seven controls aged 70–89 years with symptoms that were not severe enough for a diagnosis of AD were recruited from the Outpatient Psychosomatic Clinic of TVGH; these clinical patients suffered from depression, dementia, insomnia, or high blood pleasure. Subsequently, 154 healthy controls were recruited from the community in Taipei and National Ilan University (NIU). The total controls included individuals within the age ranges of 20–29 years (*n* = 18; men (M): 8, women (W): 10), 30–39 years (*n* = 3; M: 3, W: 0), 40–49 years (*n* = 13; M: 2, W: 11), 50–59 years (*n* = 32; M: 10, W: 22), 60–69 years (*n* = 46; M: 15, W: 31), 70–79 years (*n* = 56; M: 29, W: 27), and 80–89 years (*n* = 23; M: 16, W: 7).

A total of 47 patients who were diagnosed as having AD based on their symptoms were recruited from the Outpatient Neurology Clinic of TVGH and the Outpatient Neurology Clinic of TAH, and these patients included individuals within the age ranges of 40–49 years (n = 1; M: 0, W: 1), 50–59 years (n = 1; M: 1, W: 0), 60–69 years (*n* = 6; M: 3, W: 3), 70–79 years (*n* = 18; M: 9, W: 9), and 80–89 years (*n* = 21; M: 11, W: 10). Based on the clinical criteria for AD diagnosis involving the SPMSQ or MMSE and CT or MRI results, the AD patients were selected to form part of a clinical consensus meeting for further detection of the S-glutathionylated GAPDH levels in their blood plasma.

### Quantification of human blood S-glutathionylated GAPDH levels through ELISA

Human plasma samples were collected in collection tubes containing EDTA-2Na and centrifuged at 4000×g and 4°C for 30 min to obtain the blood protein supernatants. The supernatant samples were incubated in liquid nitrogen for more than 24 h and frozen at −80°C until analysis. We modified a previously described method [11] to develop a sandwich ELISA for detection of the concentration of S-glutathionylated GAPDH in blood plasma. The antibodies used in the developed sandwich ELISA included the following (prepared in our laboratory): rabbit polyclonal anti-human GAPDH antibody as the capture antibody, mouse monoclonal anti-GSH antibody as the primary antibody, and HRP-conjugated goat anti-mouse IgG antibody as the conjugated secondary antibody. The samples were analysed using an ELISA reader (Microplate Reader, Molecular Devices Inc., CA, USA) at a wavelength of 450 nm.

### Statistics

The quantified data on the human blood S-glutathionylated GAPDH concentrations obtained by ELISA were analysed by one-way analysis of variance (one-way ANOVA) and the Tukey–Kramer multiple-comparisons post-test (GraphPad Software Version 5.01 for Windows, GraphPad Software Inc., San Diego, CA, USA). All the data are presented as the means ± SDs, and statistical significance was set to *p* < 0.05.

We subsequently used the receiver operating characteristic (ROC) curve (MedCalc Version 13.3.1.0© 1993–2014 MedCalc Software bvba) to identify a criterion for AD diagnosis based on the blood S-glutathionylated GAPDH levels. The ROC curve is a plot of the true positive rate against the false positive rate for different possible cut-off points of a diagnostic test. The accuracy of the test depends on how well the test separates the tested group into the individuals with (represented as 1) and those without (represented as 0) the disease. The area under the ROC curve (AUC) value provides an indication of the accuracy and ranges from 1.0 to 0.5. AUC values from 0.5 to 0.7 indicate low accuracy, whereas values from 0.7 to 0.9 indicate moderate accuracy, and values greater than 0.9 indicate high accuracy. In addition, an AUC value of 0.5 indicates that the diagnostic method is completely ineffective and has no diagnostic value, and an AUC value less than 0.5 does not reflect reality or reflects a condition that rarely occurs in practice. To estimate the accuracy, AUC values with 95% CI and z statistics (*p* < 0.0001) were calculated. The ROC curve also indicates the sensitivity and specificity. The sensitivity (also called the true positive rate) constitutes a measure of the proportion of positives and is the percentage of patients with illness that were correctly identified as having the condition. The specificity (also called the true negative rate) is a measure of the proportion of negatives and is calculated as the percentage of healthy people correctly identified as not having the condition.

## Results

**Fig 1:**
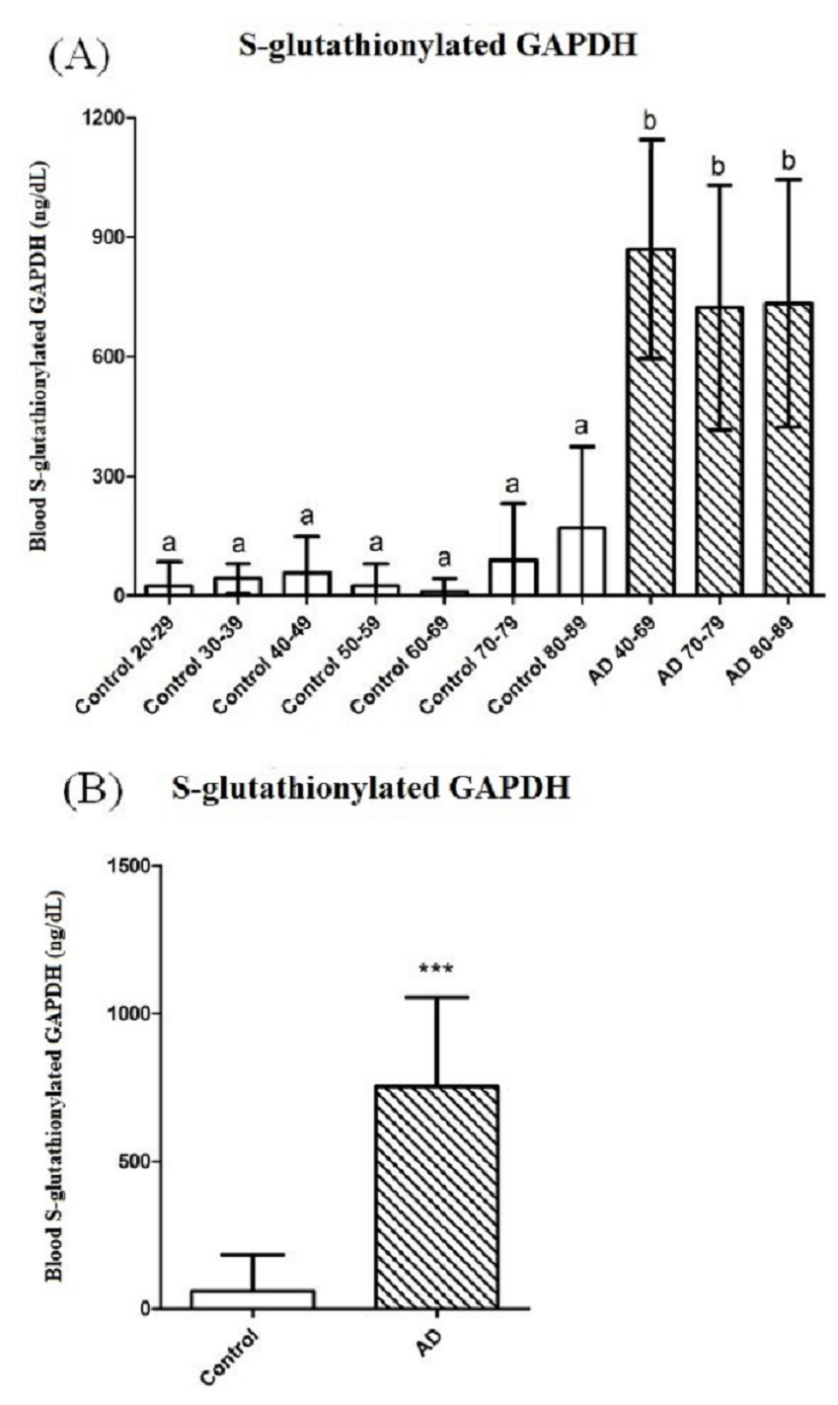
At different ages, the blood S-glutathionylated GAPDH levels of the patients with AD were higher than those of the controls. (A) The controls were separated into the age ranges of 20–29 (*n* = 18), 30–39 (*n* = 3), 40–49 (*n* = 13), 50–59 (*n* = 32), 60–69 (*n* = 46), 70–79 (*n* = 56), and 80–89 years (*n* = 23), for comparison with the patients with AD aged 40–69 (*n* = 8), 70–79 (*n* = 18), and 80–89 years (*n* = 21). ^a, b^Different letters indicate significant differences, as determined by one-way ANOVA (*p* < 0.001). (B) The comparison between the total set of controls aged 20–79 years (*n* = 191) and the total set of patients with AD aged 40–89 years (*n* = 47) showed significant differences (***p < 0.001).

The blood S-glutathionylated GAPDH data from the 191 controls, including those within the age ranges of 20–29, 30–39, 40–49, 50–59, 60–69, 70–79, and 80–89 years, were compared with those of the 47 patients with AD, including patients in the age ranges of 40–69, 70–79, and 80–89 years. According to the one-way ANOVA results, the blood S-glutathionylated GAPDH levels in patients with AD of different ages were all significantly higher than those in the controls of different ages (p < 0.001) (Fig 1A). The blood S-glutathionylated GAPDH levels in the total set of controls (59.92 ± 122.4 ng/dL) were also significantly lower than those in the total set of patients with AD (752.7 ± 301.7 ng/dL) (p < 0.001) (Fig 1B) (Table 1). No significant difference were found between different age ranges.

**Table 1:** The blood S-glutathionylated GAPDH levels were separated by gender for comparisons between the controls and the patients with AD.

Groups	No. of W^1^	S-GSH GAPDH (mean ± SD)	No of M^2^	S-GSH GAPDH (mean ± SD)	Total No.	S-GSH GAPDH (mean ± SD)
Control 80–89	7	134.0 ± 228.1^**a**^	16	185.7 ± 198.8^**a**^	23	170.0 ± 204.3^**a**^
Control 70–79	27	106.7 ± 160.0^**a**^	29	71.59 ± 125.2^**a**^	56	88.50 ± 142.8^**a**^
Control 60–69	31	14.81 ± 38.75^**a**^	15	1.33 ± 3.68^**a**^	46	10.41 ± 32.34^**a**^
Control 50–59	22	23.95 ± 51.84^**a**^	10	27.60 ± 62.84^**a**^	32	25.09 ± 54.49^**a**^
Control 40–49	11	65.18 ± 96.76^**a**^	2	14.50 ± 20.51^**a**^	13	57.38 ± 90.55^**a**^
Control 30–39	0	-	3	43.00 ± 37.40^**a**^	3	43.00 ± 37.40^**a**^
Control 20–29	8	0	10	42.22 ± 78.40^**a**^	18	23.45 ± 60.99^**a**^
Total Control	106	52.09 ± 114.8^**a**^	85	69.69 ± 131.2^**a**^	191	59.92 ± 122.4^**a**^
AD 80–89	10	758.1 ± 338.3^**b**^	11	711.2 ± 297.2^**b**^	21	733.5 ± 310.2^**b**^
AD 70–79	9	720.5 ± 306.7^**b**^	9	725.7 ± 326.0^**b**^	18	723.1 ± 307.1^**b**^
AD 40–69	4	740.5 ± 333.5^**b**^	4	998.7 ± 139.5^**b**^	8	869.6 ± 274.0^**b**^
Total AD	23	740.3 ± 310.7^**b**^	24	764.6 ± 299.0^**b**^	47	752.7 ± 301.7^**b**^

^**1,2**^ W and M represent women and men, respectively.

^**a,b**^ Different letters in the same row indicate significant differences, as determined by one-way ANOVA (*p* < 0.001).

**Fig 2:**
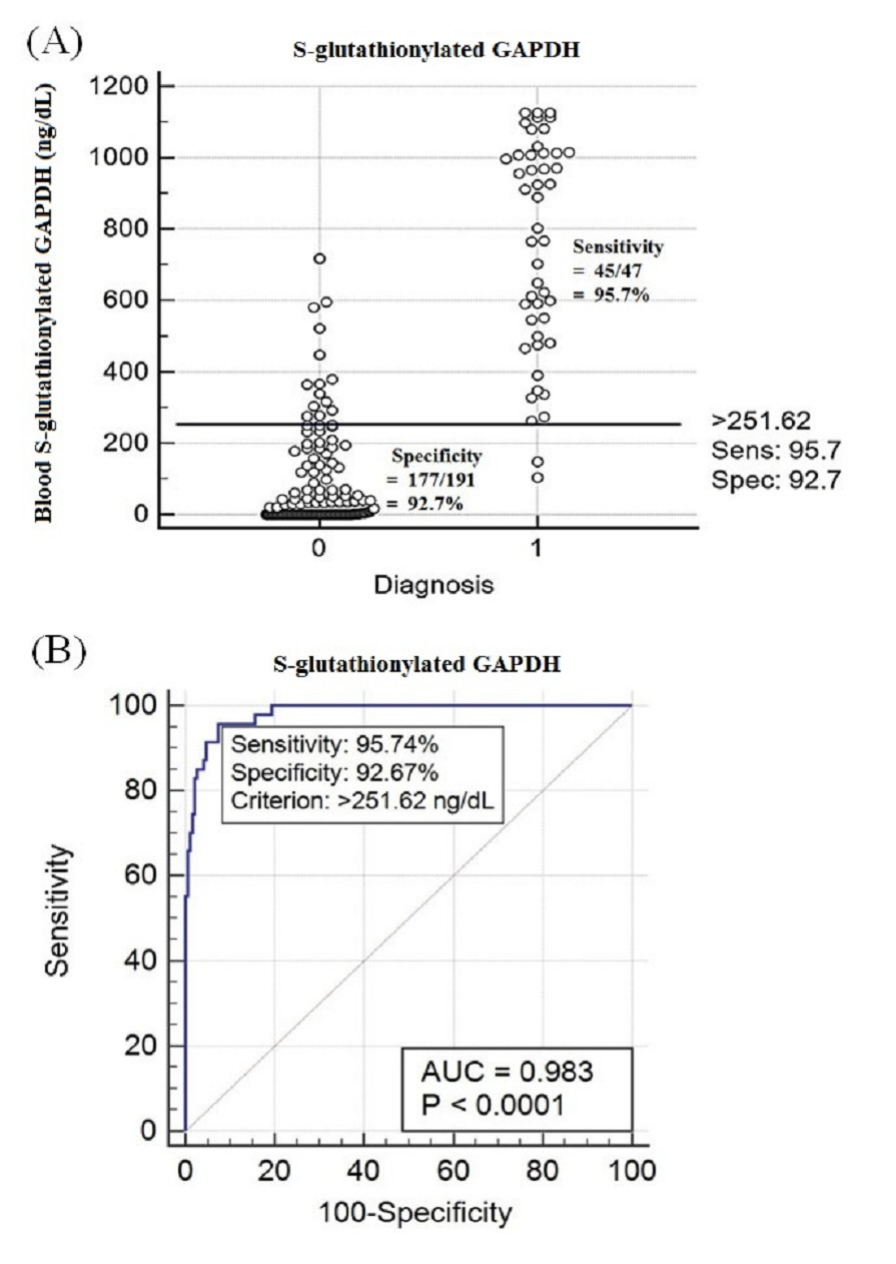
Receiver Operating Characteristic (ROC) curve used to identify the criterion based on the blood S-glutathionylated GAPDH levels for AD diagnosis. (A) The criterion was a blood S-glutathionylated GAPDH level > 251.62 ng/dL. The sensitivity was 95.74%, and the specificity was 92.67%. The numbers 0 and 1 represent the controls and patients with AD, respectively. (B) The area under the ROC curve (AUC) was 0.983 ± 0.0.00691, which indicated a high accuracy for AD diagnosis with a 95% CI of 0.957–0.995 (z statistic 69.814, *p* < 0.0001).

Based on the ROC, a blood S-glutathionylated GAPDH level > 251.62 ng/dL was defined as a criterion for AD diagnosis (Fig 2A). The sensitivity (percentage of patients with illness correctly identified as having the condition) was 95.74%, and the specificity (percentage of healthy people correctly identified as not having the condition) was 92.67%. The AUC was 0.983 ± 0.0.00691, which indicated a high accuracy for AD diagnosis with a 95% CI of 0.957–0.995 (z statistic 69.814, *p* < 0.0001) (Fig 2B). The AUC was higher than 0.9, which indicated that the criterion blood S-glutathionylated GAPDH level > 251.62 ng/dL exhibited a high accuracy for the diagnosis of AD.

**Fig 3:**
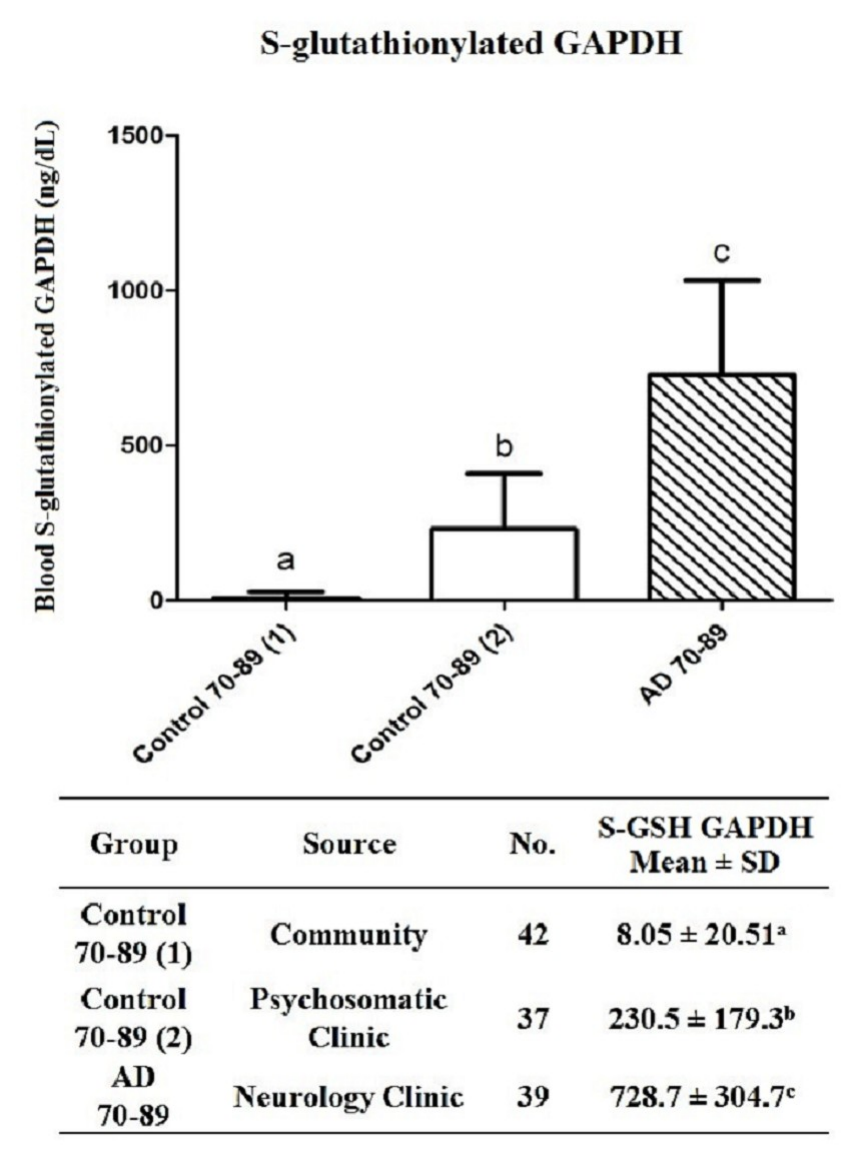
The blood S-glutathionylated GAPDH levels of the healthy controls from the community (1) are markedly lower than those of the controls from the outpatient psychosomatic clinic (2) and the patients with AD. ^a, b, c^ Different letters indicate significant differences, as determined by one-way ANOVA (*p* < 0.001).

However, the blood S-glutathionylated GAPDH levels in the 37 clinical controls aged 70–89 years from the Outpatient Psychosomatic Clinic, who suffered from depression, dementia, insomnia, or high blood pleasure, were higher (230.5 ± 179.3 ng/dL) than those in the 42 healthy controls aged 70–89 years recruited from the community (8.05 ± 20.51 ng/dL) (p < 0.001) (Fig 3). In fact, the blood S-glutathionylated GAPDH levels of these clinical controls were still lower than those of the 39 patients with AD aged 70–89 years who were recruited from the Outpatient Neurology Clinic (728.7 ± 304.7 ng/dL) (p < 0.001) (Fig 3).

Moreover, no differences were observed between women (W) and men (M) or between different ages (Table 1).

## Discussion

The glycolytic enzyme GAPDH was once considered a simple “housekeeping” protein [28, 30], but recent studies have shown that this enzyme is involved in many cellular processes in addition to glycolysis, including DNA repair [41], tRNA export [42], membrane fusion and transport [43], cytoskeletal dynamics [44], and cell death [45]. The multifunctional properties of GAPDH are regulated by its oligomerization, posttranslational modification, and subcellular localization. The multifunctional properties of GAPDH in distinct subcellular domains of the cytoplasm, vesicles, mitochondria, and nucleus might play a common biological role in the stress response.

A large amount of oxidative GAPDH has been detected in the brains of AD patients. GAPDH is no longer a simple “housekeeping” protein, and oxidative GAPDH has been identified as a major component of amyloid plaques and NFTs in the brains of patients with AD [46].

**Fig 4:**
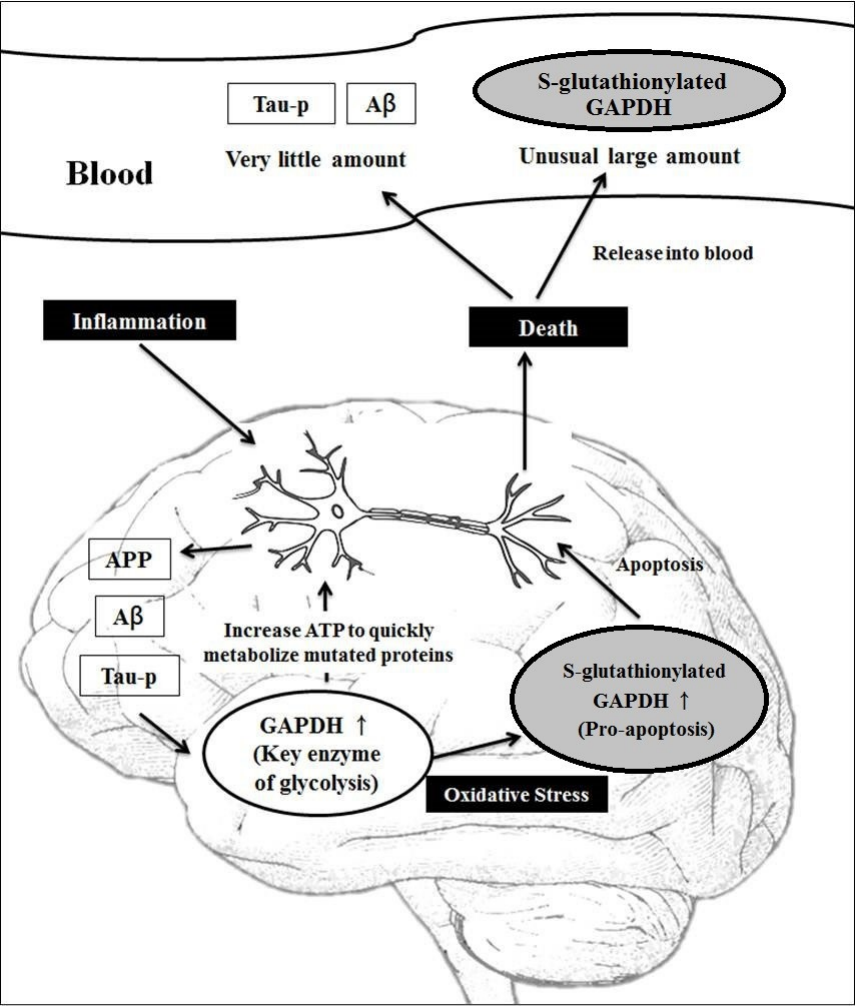
Novel pathway through which the release of blood S-glutathionylated GAPDH from apoptotic neurons reflects the extent of neuronal apoptosis in AD brains.

We propose the following novel explanation (Fig 4): when the accumulation of APP and/or Aβ occurs, GAPDH might play an important role in ensuring neuron survival. The normal GAPDH levels in the cytosol first increase to enhance the ATP levels through glycolysis. After it translocates into the nucleus, GAPDH participates in the initiation of apoptosis and the transcription of genes involved in anti-apoptotic pathways and cell proliferation and plays a role in the regulation of telomere length [41]. Additionally, GAPDH stimulates the autophagy-mediated clearance of apoptotic mitochondria [40] and participates in the regulation of mRNA binding and stability [20]. However, the increasing accumulation of APP and/or Aβ in disorders binds to GAPDH and causes GAPDH oxidation. Furthermore, the p300/CREB-binding protein acetylates oxidative GAPDH to activate downstream p53. In contrast, oxidative GAPDH induces mitochondrial apoptosis and neuronal death [47, 48]. After neuron death, large amounts of S-glutathionylated GAPDH are likely released into the blood plasma.

In fact, a previous report confirmed that GAPDH, when it accumulates in mitochondria to alter the permeabilization of the mitochondrial membrane, has the ability to induce cell apoptosis and can thus be considered a proapoptotic factor [24]. Furthermore, oxidative GAPDH plays an apparent role in AD-related apoptotic cell death [25].

Initially, GAPDH exists in the cytoplasm rather than in the blood, and GAPDH is not detected in the blood plasma of normal individuals but is detected at significant levels in the blood plasma of patients with AD. We hypothesize that S-glutathionylated GAPDH is released into the blood plasma when neurons start to die due to AD. For this reason, we tested the S-glutathionylated GAPDH levels in the blood plasma of AD patients. Increased levels of blood S-glutathionylated GAPDH could represent the level of neuronal apoptosis under the current conditions. A higher level of blood S-glutathionylated GAPDH indicates a more serious level of neuronal apoptosis in the brain. If the apoptotic conditions continue for a long time, progression to AD becomes more possible.

In this study, the blood S-glutathionylated GAPDH levels were positively correlated with AD, irrespective of age and gender. Based on the ROC statistics, a criterion of blood S-glutathionylated GAPDH levels > 251.62 ng/dL showed high accuracy for the diagnosis of AD (0.983 ± 0.0.00691 of AUC). Thus, if a patient’s blood S-glutathionylated GAPDH level is higher than 251.62 ng/dL, the patient is at high risk of developing AD in the future (Fig 2).

The blood S-glutathionylated GAPDH levels of patients with AD had to reach least 752.7 ± 301.7 ng/dL before a diagnosis could be made through comprehensive examinations involving MMSE, CT, and/or MRI at the Outpatient Neurology Clinic (Table 1). However, the process of brain neuronal apoptosis should begin at a much earlier stage. Specifically, we found that the S-glutathionylated GAPDH levels in the blood plasma of both control groups at the age of 70–89 years, namely, 37 patients from the psychosomatic clinic and 42 healthy persons, showed significant blood S-glutathionylated GAPDH levels of 230.5 ± 179.3 and 8.05 ± 20.51 ng/dL, respectively (Fig 3). Because some studies have shown that patients with severe depression and apathy are at high risk for subsequent dementia or AD [49, 50], we hypothesized that these levels might indicate the occurrence of neurodegenerative AD progression in psychosomatic patients.

To date, the correlation between blood S-glutathionylated GAPDH levels and AD progression has not been studied, and this study provides the first investigation of this correlation. The results of this study suggest that the development of a marker to estimate the degree of neuronal apoptosis in AD based on the blood level of S-glutathionylated GAPDH deserves further attention.

## Appendix

The institutional review board (IRB) at each participating centre (Taipei Veterans General Hospital IRB (IRB No. 2013-04-044B) and Taiwan Adventist Hospital IRB (IRB No. 108-E-10)) approved the study protocol used to investigate whether blood GAPDH could be a biomarker of Alzheimer’s disease.

## Supporting information

S1 Dataset Minimal data set. (DOCX)
