# Supplementary material for: An investigation of the correlation between the S-glutathionylated GAPDH levels in blood and Alzheimer’s disease progression
Source: PLoS One. 2020 May 29;15(5):e0233289. doi: 10.1371/journal.pone.0233289 (PMC7259681; doi:10.1371/journal.pone.0233289)
Supplement: S1 Dataset — (DOCX) [file pone.0233289.s001.docx]

**Minimal data set**

| Fig. # | Mean | S.D | S.E | Statistical method used | P value | # sample |
| --- | --- | --- | --- | --- | --- | --- |
| Fig. 1A |  |  |  | Aaccording to the one-way analysis of variance, through the Tukey–Kramer multiple comparisons post-test | P < 0.001 |  |
| Control 20-29 | 23.45 | 60.99 | 14.38 |  |  | 18 |
| Control 30-39 | 43.00 | 37.40 | 21.59 |  |  | 3 |
| Control 40-49 | 57.38 | 90.55 | 25.11 |  |  | 13 |
| Control 50-59 | 25.09 | 54.49 | 9.633 |  |  | 32 |
| Control 60-69 | 10.41 | 32.34 | 4.768 |  |  | 46 |
| Control 70-79 | 88.50 | 142.8 | 19.09 |  |  | 56 |
| Control 80-89 | 170.0 | 204.3 | 42.60 |  |  | 23 |
| AD 40-69 | 869.6 | 274.0 | 96.86 |  |  | 8 |
| AD 70-79 | 723.1 | 307.1 | 72.38 |  |  | 18 |
| AD 80-89 | 733.5 | 310.2 | 67.70 |  |  | 21 |
| Fig. 1B |  |  |  | Aaccording to the one-way analysis of variance, through the Tukey–Kramer multiple comparisons post-test | *** P < 0.001 |  |
| Control | 59.92 | 122.4 | 8.853 |  |  | 191 |
| AD | 752.7 | 301.7 | 44.00 |  |  | 47 |
|  |  |  |  |  |  |  |
| Fig. 3 |  |  |  | Aaccording to the one-way analysis of variance, through the Tukey–Kramer multiple comparisons post-test | P < 0.001 |  |
| Control 70-89(1) | 8.048 | 20.51 | 3.164 |  |  | 42 |
| Control 70-89(2) | 230.5 | 179.3 | 29.48 |  |  | 37 |
| AD 70-89 | 728.7 | 304.7 | 48.80 |  |  | 39 |
|  |  |  |  |  |  |  |

Fig. 2

Control (Blood S-glutathionylated GAPDH ng/dL)

| No. | ng/dL | No. | ng/dL | No. | ng/dL | No. | ng/dL | No. | ng/dL | No. | ng/dL | No. | ng/dL |
| --- | --- | --- | --- | --- | --- | --- | --- | --- | --- | --- | --- | --- | --- |
| 1 | 0 | 31 | 0 | 61 | 202 | 91 | 0 | 121 | 38.08 | 151 | 0 | 181 | 144.25 |
| 2 | 0 | 32 | 178 | 62 | 0 | 92 | 2 | 122 | 130.95 | 152 | 12 | 182 | 277.76 |
| 3 | 0 | 33 | 29 | 63 | 35 | 93 | 0 | 123 | 717.96 | 153 | 119 | 183 | 6.84 |
| 4 | 0 | 34 | 0 | 64 | 0 | 94 | 54 | 124 | 275.54 | 154 | 245.46 | 184 | 249.31 |
| 5 | 0 | 35 | 38 | 65 | 0 | 95 | 0 | 125 | 231.46 | 155 | 363.96 | 185 | 48.27 |
| 6 | 0 | 36 | 0 | 66 | 6 | 96 | 0 | 126 | 69.04 | 156 | 366.09 | 186 | 208.62 |
| 7 | 0 | 37 | 0 | 67 | 0 | 97 | 1 | 127 | 36.73 | 157 | 184.06 | 187 | 233.46 |
| 8 | 0 | 38 | 0 | 68 | 0 | 98 | 0 | 128 | 378.74 | 158 | 251.62 | 188 | 14.68 |
| 9 | 8.19 | 39 | 0 | 69 | 49 | 99 | 0 | 129 | 303.8 | 159 | 0 | 189 | 157.69 |
| 10 | 193.93 | 40 | 0 | 70 | 0 | 100 | 0 | 130 | 125.62 | 160 | 338.14 | 190 | 169.88 |
| 11 | 185.03 | 41 | 0 | 71 | 0 | 101 | 0 | 131 | 70.47 | 161 | 594.65 | 191 | 521.33 |
| 12 | 0 | 42 | 1 | 72 | 51 | 102 | 0 | 132 | 446.68 | 162 | 250.14 |  |  |
| 13 | 0 | 43 | 68 | 73 | 0 | 103 | 0 | 133 | 0 | 163 | 0 |  |  |
| 14 | 0 | 44 | 0 | 74 | 0 | 104 | 0 | 134 | 0 | 164 | 0 |  |  |
| 15 | 0 | 45 | 0 | 75 | 0 | 105 | 0 | 135 | 8 | 165 | 5 |  |  |
| 16 | 0 | 46 | 0 | 76 | 0 | 106 | 14 | 136 | 17 | 166 | 0 |  |  |
| 17 | 0 | 47 | 0 | 77 | 0 | 107 | 2 | 137 | 0 | 167 | 0 |  |  |
| 18 | 35 | 48 | 0 | 78 | 0 | 108 | 0 | 138 | 0 | 168 | 0 |  |  |
| 19 | 0 | 49 | 119 | 79 | 0 | 109 | 4 | 139 | 33 | 169 | 0 |  |  |
| 20 | 68 | 50 | 0 | 80 | 0 | 110 | 0 | 140 | 21 | 170 | 0 |  |  |
| 21 | 61 | 51 | 0 | 81 | 0 | 111 | 0 | 141 | 9 | 171 | 0 |  |  |
| 22 | 35 | 52 | 98 | 82 | 88 | 112 | 0 | 142 | 1 | 172 | 3 |  |  |
| 23 | 0 | 53 | 0 | 83 | 0 | 113 | 0 | 143 | 0 | 173 | 0 |  |  |
| 24 | 293 | 54 | 198 | 84 | 3 | 114 | 0 | 144 | 3 | 174 | 0 |  |  |
| 25 | 44 | 55 | 1 | 85 | 0 | 115 | 0 | 145 | 28 | 175 | 0 |  |  |
| 26 | 0 | 56 | 4 | 86 | 1 | 116 | 0 | 146 | 42 | 176 | 0 |  |  |
| 27 | 137 | 57 | 33 | 87 | 189 | 117 | 42.3 | 147 | 0 | 177 | 0 |  |  |
| 28 | 0 | 58 | 0 | 88 | 0 | 118 | 579.68 | 148 | 0 | 178 | 0 |  |  |
| 29 | 30 | 59 | 0 | 89 | 0 | 119 | 316.18 | 149 | 1 | 179 | 3 |  |  |
| 30 | 0 | 60 | 0 | 90 | 21 | 120 | 138.09 | 150 | 0 | 180 | 33 |  |  |

AD (Blood S-glutathionylated GAPDH ng/dL)

| No. | ng/dL | No. | ng/dL |
| --- | --- | --- | --- |
| 1 | 767 | 31 | 1113.82 |
| 2 | 802.77 | 32 | 1014 |
| 3 | 1006.86 | 33 | 765 |
| 4 | 925.02 | 34 | 911.12 |
| 5 | 263 | 35 | 1126.56 |
| 6 | 1112.57 | 36 | 589.92 |
| 7 | 1082.35 | 37 | 592.28 |
| 8 | 997.08 | 38 | 648.73 |
| 9 | 498.86 | 39 | 1080.54 |
| 10 | 465.12 | 40 | 1012.98 |
| 11 | 965.8 | 41 | 888.78 |
| 12 | 1125.77 | 42 | 390.88 |
| 13 | 599.56 | 43 | 337.08 |
| 14 | 475.78 | 44 | 955.42 |
| 15 | 927.11 | 45 | 273.36 |
| 16 | 1098.39 | 46 | 1031.19 |
| 17 | 328.18 | 47 | 612 |
| 18 | 1126.84 |  |  |
| 19 | 103.61 |  |  |
| 20 | 702.35 |  |  |
| 21 | 1008.34 |  |  |
| 22 | 622.59 |  |  |
| 23 | 971.32 |  |  |
| 24 | 545.85 |  |  |
| 25 | 969 |  |  |
| 26 | 481.53 |  |  |
| 27 | 551.13 |  |  |
| 28 | 147.08 |  |  |
| 29 | 1014.51 |  |  |
| 30 | 347.46 |  |  |

# ROC curve

| Variable | Test |
| --- | --- |
| Classification variable | Diagnosis |

| Sample size | 238 |
| --- | --- |
| Positive group ^a^ | 47 (19.75%) |
| Negative group ^b^ | 191 (80.25%) |

^a^ Diagnosis = 1
^b^ Diagnosis = 0

Area under the ROC curve (AUC)

| Area under the ROC curve (AUC) | 0.983 |
| --- | --- |
| Standard Error ^a^ | 0.00691 |
| 95% Confidence interval ^b^ | 0.957 to 0.995 |
| z statistic | 69.814 |
| Significance level P (Area=0.5) | <0.0001 |

^a^ DeLong et al., 1988

^b^ Binomial exact

## Youden index

| Youden index J | 0.8841 |
| --- | --- |
| Associated criterion | >251.62 |
| Sensitivity | 95.74 |
| Specificity | 92.67 |

## Criterion values and coordinates of the ROC curve [[Show]](javascript:showdiv('d0','d1','table1');)

| Criterion | Sensitivity | 95% CI | Specificity | 95% CI | +LR | -LR |
| --- | --- | --- | --- | --- | --- | --- |
| ≥0 | 100.00 | 92.5 - 100.0 | 0.00 | 0.0 - 1.9 | 1.00 |  |
| >98 | 100.00 | 92.5 - 100.0 | 80.63 | 74.3 - 86.0 | 5.16 | 0.00 |
| >103.61 | 97.87 | 88.7 - 99.9 | 80.63 | 74.3 - 86.0 | 5.05 | 0.026 |
| >144.25 | 97.87 | 88.7 - 99.9 | 84.29 | 78.3 - 89.1 | 6.23 | 0.025 |
| >147.08 | 95.74 | 85.5 - 99.5 | 84.29 | 78.3 - 89.1 | 6.10 | 0.050 |
| >251.62 | 95.74 | 85.5 - 99.5 | 92.67 | 88.0 - 95.9 | 13.06 | 0.046 |
| >273.36 | 91.49 | 79.6 - 97.6 | 92.67 | 88.0 - 95.9 | 12.48 | 0.092 |
| >316.18 | 91.49 | 79.6 - 97.6 | 95.29 | 91.2 - 97.8 | 19.42 | 0.089 |
| >337.08 | 87.23 | 74.3 - 95.2 | 95.29 | 91.2 - 97.8 | 18.51 | 0.13 |
| >338.14 | 87.23 | 74.3 - 95.2 | 95.81 | 91.9 - 98.2 | 20.83 | 0.13 |
| >347.46 | 85.11 | 71.7 - 93.8 | 95.81 | 91.9 - 98.2 | 20.32 | 0.16 |
| >378.74 | 85.11 | 71.7 - 93.8 | 97.38 | 94.0 - 99.1 | 32.51 | 0.15 |
| >390.88 | 82.98 | 69.2 - 92.4 | 97.38 | 94.0 - 99.1 | 31.70 | 0.17 |
| >446.68 | 82.98 | 69.2 - 92.4 | 97.91 | 94.7 - 99.4 | 39.62 | 0.17 |
| >498.86 | 74.47 | 59.7 - 86.1 | 97.91 | 94.7 - 99.4 | 35.56 | 0.26 |
| >521.33 | 74.47 | 59.7 - 86.1 | 98.43 | 95.5 - 99.7 | 47.41 | 0.26 |
| >551.13 | 70.21 | 55.1 - 82.7 | 98.43 | 95.5 - 99.7 | 44.70 | 0.30 |
| >579.68 | 70.21 | 55.1 - 82.7 | 98.95 | 96.3 - 99.9 | 67.05 | 0.30 |
| >592.28 | 65.96 | 50.7 - 79.1 | 98.95 | 96.3 - 99.9 | 62.99 | 0.34 |
| >594.65 | 65.96 | 50.7 - 79.1 | 99.48 | 97.1 - 100.0 | 125.98 | 0.34 |
| >702.35 | 55.32 | 40.1 - 69.8 | 99.48 | 97.1 - 100.0 | 105.66 | 0.45 |
| >717.96 | 55.32 | 40.1 - 69.8 | 100.00 | 98.1 - 100.0 |  | 0.45 |
| >1126.84 | 0.00 | 0.0 - 7.5 | 100.00 | 98.1 - 100.0 |  | 1.00 |
